# *Curcumae Rhizoma* - combined with *Sparganii Rhizoma* in the treatment of liver cancer: Chemical analysis using UPLC-LTQ-Orbitrap MS^n^, network analysis, and experimental assessment

**DOI:** 10.3389/fphar.2022.1027687

**Published:** 2022-12-05

**Authors:** Jing Wei, Xiaoping Wang, Ying Dong, Xiangjian Zhong, Xueyang Ren, Ruolan Song, Jiamu Ma, Axiang Yu, Qiqi Fan, Jianling Yao, Dongjie Shan, Fang Lv, Yuan Zheng, Qingyue Deng, Xianxian Li, Yingyu He, Shusheng Fan, Chongjun Zhao, Xiuhuan Wang, Ruijuan Yuan, Gaimei She

**Affiliations:** ^1^ School of Chinese Materia Medica, Beijing University of Chinese Medicine, Beijing, China; ^2^ Beijing Key Laboratory for Quality Evaluation of Chinese Materia Medica, Beijing, China; ^3^ State Key Laboratory of Natural Medicines, New Drug Screening Center, Jiangsu Center for Pharmacodynamics Research and Evaluation, China Pharmaceutical University, Nanjing, China; ^4^ Beijing Huilongguan Hospital, Peking University HuiLongGuan Clinical Medical School, Beijing, China

**Keywords:** *Curcumae Rhizoma* - *Sparganii Rhizoma*, liver cancer, molecular networking, UPLC-LTQ-Orbitrap MS^n^, zebrafish xenograft model

## Abstract

**Objective:** *Curcumae Rhizoma*–*Sparganii Rhizoma* (CR-SR) is a traditional botanical drug pair that can promote blood circulation, remove blood stasis, and treat tumors in clinics. The aim of the present study was to investigate the therapeutic material basis and potential mechanisms of CR-SR, CR, and SR for the treatment of liver cancer.

**Method:** The chemical profile analyses of CR-SR, CR, and SR were performed by molecular networking and UPLC-LTQ-Orbitrap MS^n^. The anti-liver cancer activities of CR-SR, CR, and SR were assessed by using a zebrafish xenograft model *in vivo* for the first time and detected by the HepG2 cell model *in vitro*. Combining the network analysis and molecular docking, real-time quantitative polymerase chain reaction (RT-qPCR) experiments were undertaken to further explore the mechanisms of CR-SR, CR, and SR for the treatment of liver cancer.

**Results:** In total, 65 components were identified in CR-SR, CR, and SR. Based on the clusters of molecular networking, a total of 12 novel diarylheptanoids were identified from CR-SR and CR. By combining our results with information from the literature, 32 sesquiterpenoids and 21 cyclic dipeptides were identified from CR-SR, CR, and SR. The anti-liver cancer activities were observed in both the drug pair and the single botanical drugs *in vitro* and *in vivo*, and the order of activity was CR-SR > CR > SR. They could downregulate the expression of proto-oncogene tyrosine-protein kinase Src (SRC), epidermal growth factor receptor (EGFR), estrogen receptor-α (ESR1), prostaglandin endoperoxide synthase 2 (PTGS2), and amyloid precursor protein (APP).

**Conclusion:** Taken together, the present study provided an experimental basis for the therapeutic material basis and potential molecular mechanisms of CR-SR, CR, and SR. This study provided a novel insight for objective clinical treatment of liver cancer.

## 1 Introduction

A drug pair in traditional Chinese medicine (TCM) refers to two botanical drugs that exhibit synergistic pharmaceutical and/or detoxification activities, playing an important role in the exploration of general botanical drug compatibility (Wang et al., 2012; Yu et al., 2019). The compatibility of CR and SR is a traditional drug pair for promoting blood circulation and removing blood stasis in clinics, which shows a tendency to reinforce each other (Xu et al., 2015). According to traditional Chinese medicine, activating blood circulation and removing blood stasis could dredge the meridians, improve microcirculation, and adjust and strengthen immune function to dampen cancer and shrink the lump (Lu and Li, 2009). Modern pharmacological studies have shown that the occurrence and development of tumors are related to angiogenesis and the abnormal blood coagulation system. The current research and clinical practice mostly focus on anti-liver cancer activities (Xu et al., 2015).

Our team has been committed to studying the anti-cancer activities of CR-SR, CR, and SR. The results showed their broad-spectrum anti-tumor activity *in vitro*. Also, the activity of the drug pair was better than the single drug (Liu et al., 2021). The anti-liver cancer activity *in vivo* of CR-SR, CR, and SR is still obscure. Zebrafish have been widely used in tumor research because they have highly conserved oncogenes, tumor suppressor genes, and cell cycle regulatory genes with humans. The features of zebrafish in rapid cell division and delayed apoptosis in embryos are similar to those of human tumors (Lu et al., 2011; Shuo et al., 2018). Therefore, zebrafish can test activity evaluation and gene expression in mechanism research.

The linear diarylheptanoids of CR-SR, CR, and SR were determined by the UPLC-MS method in previous research (Chang et al., 2020). Molecular networking is a technique used for rapid and massive identification of known compounds, similar compounds, and discovering new compounds in recent years (Le Daré et al., 2021; Zhao et al., 2021). The combination of LC-MS and molecular networking can make the analysis of MS data less time-consuming and difficult.

Network analysis is a very promising approach for finding potential drug targets (Tao et al., 2013). Constructing a multi-level network through omics data analysis and computer simulation methods provides a new idea for the systematic research of the complex system of TCM (Li and Zhang, 2013). Molecular docking is a method of designing drugs by simulating the interaction between receptors and drugs and plays an important role in revealing the mechanism of action between active components and body targets (Yuan et al., 2021). It is a common strategy to explore the potential mechanism of action of drugs on diseases by combining network analysis and molecular docking.

**FIGURE 1:**
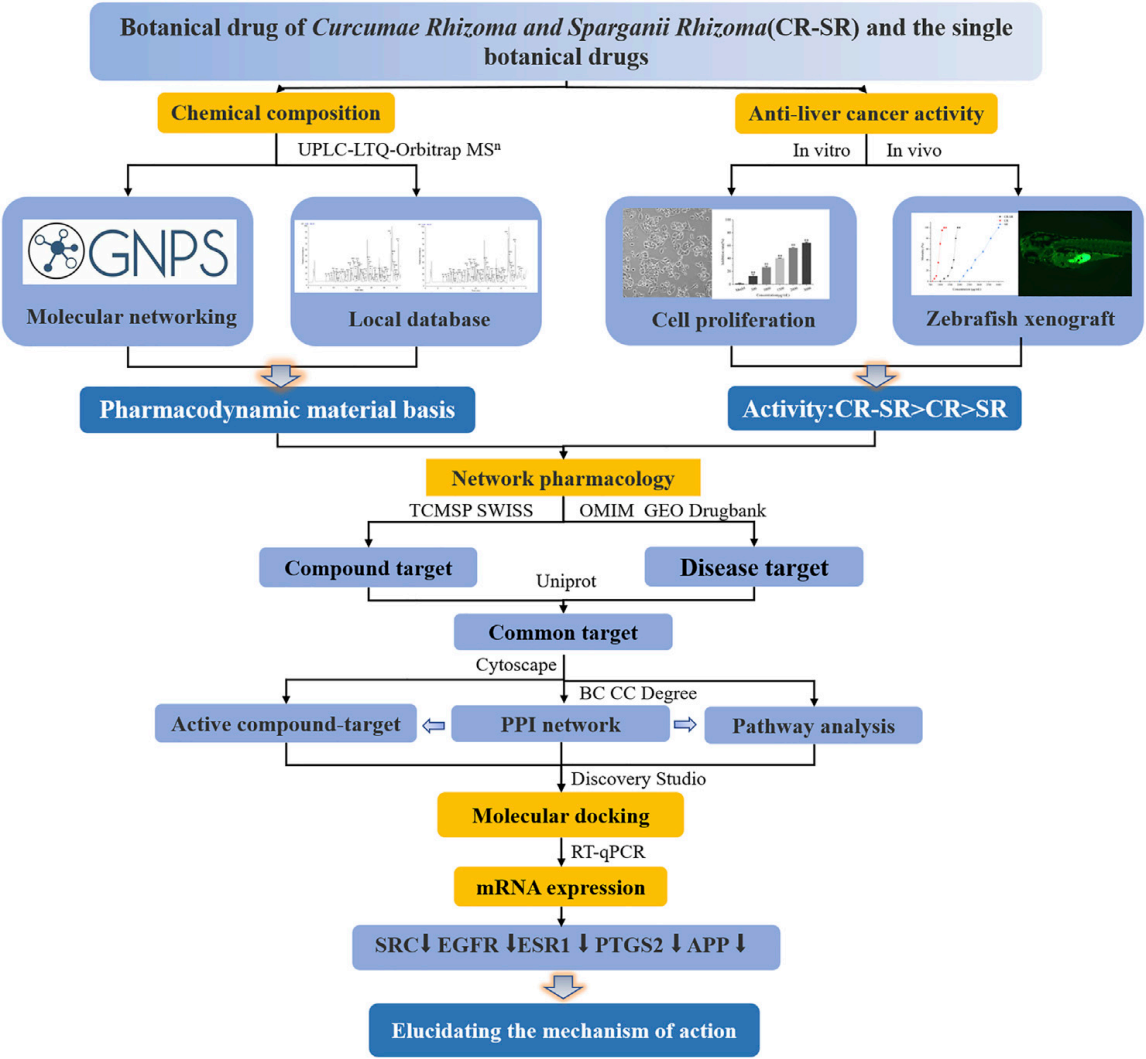
Flow chart of the study process.

The introduction of molecular networking provided an opportunity to discover novel compounds in this study. The novel diarylheptanoids were deduced from a cluster of the network map structures of molecular networking. At the same time, the chemical components of CR-SR, CR, and SR water extracts were subjected to a comprehensive analysis. The cyclic dipeptide and sesquiterpenoid components were analyzed in detail using UPLC-LTQ-Orbitrap MS^n^ technology. The zebrafish xenograft HepG2 model was used for the first time to study the anti-liver cancer activities of CR-SR, CR, and SR *in vivo,* and the mechanism of action was explored by combining network pharmacology, molecular docking, and RT-qPCR technology. This study provided a solid foundation on the therapeutic material basis and mechanism of action for the anti-liver cancer activities of CR-SR, CR, and SR. A flow chart of the study process is shown in Figure 1.

## 2 Materials and methods

### 2.1 General experimental procedures

The UPLC-MS was carried out on a Thermo Dionex UltiMate 3000 system (Thermo Fisher Scientific, United States ) using an Agilent XDB-C_18_ column (4.6 × 150 mm, 3.6 μm) and a Thermo LTQ-Orbitrap Velos Pro Hybrid (Thermo Fisher Scientific, United States ) equipped with an ESI source operating in the auto-MS^n^ mode. Trichloromethane, isopropyl alcohol, and anhydrous ethanol were provided by Fuchen Chemical Reagent Co., Ltd. (Tianjin, China). NaCl, KCl, CaCl_2_, NaHCO_3_, Na_2_HPO_4_, KH_2_PO_4_, and MgSO_4_.7H_2_O (analytical reagent grade) were purchased from Beijing Chemical Plant Co., Ltd. (Beijing, China). RPMI 1640 and fetal bovine serum (FBS) were ordered from Biological Industries. Pancreatin was obtained from Servicebio. Penicillin and streptomycin were purchased from Shanghai Yuanye Biotechnology Co., Ltd. (Beijing, China). Cell-Counting Kit-8 was purchased from LABLEAD. DiO fluorescent dye was purchased from Beyotime Biotechnology (Beyotime, China). The tissue Total RNA Isolation Kit, RNase free, 4 × gDNA wiper Mix, 5 × HiScript III qRT SuperMix, ChamQ SYBR Color qPCR Master Mix, and HiScript III-RT SuperMix for qPCR were purchased from Vazyme Biotech Co., Ltd. (Nanjing, China).

### 2.2 Plant material

CR and SR were provided by Hebei Anguo Medical Materials Corporation (Anguo, China) and identified as *Curcuma kwangsiensis* S.G. Lee et C.F. Liang and *Sparganium stoloniferum* Buch.-Ham., respectively, by Professor Jing-Juan Wang from the Beijing University of Chinese Medicine, China. The voucher specimens (20180327 and 170201001) were stored in laboratory B417 at the Beijing University of Chinese Medicine.

### 2.3 Extraction

The botanical drug pair (200 g, CR-SR = 1:1) was decocted with deionized water three times for 2 h each. Then, the supernatant was concentrated under reduced pressure, and finally, a dried extract was obtained. The single botanical drugs CR (200 g) and SR (200 g) were treated in the same way.

### 2.4 Chemical profiling

#### 2.4.1 Molecular networking analysis

All instruments were controlled by the Xcalibur data system. The data acquired from the previous research were carried out by analyst software Xcalibur 2.1 (Thermo Fisher Scientific, Bremen, Germany) (Chang et al., 2020). The mass spectral data were converted from the raw format to the mzXML format using the msConvert. Then, the file was uploaded to the Global Natural Products Social Networking (GNPS) analysis platform (https://gnps.ucsd.edu) to build a molecular network (Xue et al., 2021). In the (GNPS) web-based platform, the basic parameters were modified to *m/z* 0.02 for the mass tolerance of precursor and fragment ions used in the MS/MS spectral library. Furthermore, the cosine fraction threshold was set to 0.6, and the results were visualized by Cytoscape 3.8.2 software.

#### 2.4.2 UPLC-LTQ-Orbitrap MS^n^ analysis

The separation of analytes was achieved using the gradient elution system consisting of acetonitrile (A) and 0.05% aqueous formic acid (B) at a flow rate of 0.3 ml/min. The gradient program was as follows: 0–5 min, 3%–12% A; 5–11 min, 12%–20% A; 11–14 min, 20%–25% A; 14–17 min, 25%–30% A; 17–19 min, 30% A; 19–20 min, 30%–35% A; 20–22 min, 35% A; 22–25 min, 35%–40% A; 25–28 min, 40%–48% A; 28–30 min, 48%–52% A; 30–35 min, 52%–80% A; and 35–40 min, 80% A. Then, 1 µL aliquot of each sample was injected into the column, which was maintained at 35°C.

Data acquisition of the mode scan was performed from *m/z* 50 to *m/z* 1000 at a resolution of 30,000 in both positive and negative modes. The following ESI parameters in the negative ion mode were optimized and used: a capillary temperature of 350°C, sheath gas flow rate of 40 arb, auxiliary gas flow rate of 10 arb, electrospray voltage of −3.5 V, and tube lens voltage of −120 V. The electrospray voltage was 3.4 V, and the tube lens voltage was 120 V in the positive ion mode. Other parameters were the same as those of the negative ion mode. The most intense ions detected in the full-scan spectrum were selected for the data-independent scan. The relative collision energy for collision-induced dissociation was set to 35% of the maximum.

### 2.5 Network analysis and molecular docking

#### 2.5.1 Target prediction

The compounds collected from the identification of chemical components by molecular networking and UPLC-MS^n^ analysis included 59 diarylheptanoids, 32 sesquiterpenoids, 21 cyclic dipeptides, and 40 phenols and organic acids (Chang et al., 2020; Wang et al., 2020). The active compounds were further screened based on oral bioavailability (OB) ≥ 30 and drug-like properties (DL) ≥ 0.18, and their predicted targets were obtained by TCM Systems Pharmacology (TCMSP) (https://old.tcmsp-e.com/tcmsp.php) and the Swiss target prediction database (http://swisstargetprediction.ch/). The disease targets were collected by searching for the keywords “liver cancer” in the Gene Expression Omnibus database (https://www.ncbi.nlm.nih.gov/geo/), DrugBank (https://go.drugbank.com/), and Online Mendelian Inheritance in Man (OMIM, http://www.omim.org/). All predicted targets were standardized into official gene symbols using the UniProt database (https://www.uniprot.org/).

#### 2.5.2 Network construction

Network construction was performed as follows: 1) the active compound–target network; 2) protein–protein interaction (PPI) network. We constructed a Venn diagram to determine the overlapping targets between the active compound targets and disease targets using Venny online (https://bioinfogp.cnb.csic.es/tools/venny/). These overlapping targets might play an important role when CR-SR, CR, and SR treat liver cancer. The intersection was considered as the potential targets and inputted to the STRING (https://string-db.org/) database to construct the relationship between proteins. The TSV format of the PPI network was downloaded and visualized by Cytoscape 3.8.2 software (Lan et al., 2020).

#### 2.5.3 Pathway enrichment

The key targets were put into the Metascape database, and the species were set as “*Homo sapiens*,” with *p* < 0.01 from the results of PPI analysis. GO annotations of targets and KEGG pathways were enriched and analyzed through the Metascape (http://metascape.org) database platform, which could analyze the biological processes and pathways of genes (Yu et al., 2021). In addition, the correspondence was established among active compounds, key targets, and the top 20 pathways of the KEGG pathway enrichment analysis results and visualized by Cytoscape 3.8.2 software.

#### 2.5.4 Molecular docking

The docking was carried out by the CDOCKER module in the Discovery Studio 2016 package. The key targets were imported into the Protein Data Bank (https://www.rcsb.org/) database to download protein in the PDB format. The 3D structures of the active compounds downloaded from the PubChem database (https://pubchem.ncbi.nlm.nih.gov/) were dealt with ChemDraw software. The docking results were evaluated with a threshold of 80% docking score for the original ligand and receptor after determining the docking pocket coordinates. Generally, the binding capacity is stronger when the docking score values are higher than the threshold value (Yuan et al., 2021).

### 2.6 Cell culture and zebrafish husbandry

The HepG2 cell line was purchased from the Cell Resource Center, Institute of Basic Medical Sciences, Chinese Academy of Medical Sciences, and Peking Union Medical College, Beijing, China. The cells were maintained in RPMI 1640 with 10% fetal bovine serum, 1% penicillin, and 1% streptomycin at 37°C in an air–5% CO_2_ incubator at constant humidity (Chen et al., 2018).

The wild-type (AB stain) zebrafish was purchased from the laboratory of Bo Zhang at Peking University. The zebrafish were maintained and raised in a continuous flow system (ESEN, Beijing, China) on a 14-h light/10-h dark cycle at 28.5°C and fed brine shrimp three times daily. The zebrafish embryos were obtained from spawning adults with a sex ratio of 1:1 (5–8 months old) and raised at 28.5°C in embryo water (5.4 mmol/L KCl, 0.137 mol/L NaCl, 1.3 mmol/L CaCl_2_, 0.25 mmol/L Na_2_HPO4, 0.44 mmol/L K_2_HPO_4_, 1.0 mmol/L MgSO_4_, and 4.2 mmol/L NaHCO_3_) (Zhao et al., 2020)_._

### 2.7 CCK8 for cell proliferation detection

Cells in the logarithmic growth phase were seeded into a 96-well plate at a density of 4 × 10^4^/ml (100 μL/well) and cultured for 24 h. Cells treated with the medium containing 0.1% DMSO were used as a negative control. CR-SR, CR, and SR extracts were well dissolved in the medium containing 0.1% DMSO, respectively, followed by ultrasonic vibration. The samples (100 μL) of CR-SR, CR, and SR at different concentrations were added to adherent cells with three wells for each group and repeated three times. After 48 h, the fluid was removed, and CCK8 (100 μL) was added to each well and cultured for 2 h. The absorbance was measured at 450 nm in a microplate reader (Beijing Perlong New Technology Co., Ltd., Beijing, China). The data were expressed as percentage inhibition compared with a vehicle (DMSO) control (Chen et al., 2018).

### 2.8 Anti-liver cancer activity of CR-SR, CR, and SR extracts in a zebrafish HepG2 xenograft model

To determine the maximum non-lethal concentration (MNLC) of these drugs, 3 days postfertilization (3 dpf) zebrafish were treated with the testing drugs in 12-well culture plates (20 larvae/well) for 72 h, and mortality was recorded at the end of treatment. The dead larvae were counted daily. Therefore, HepG2 cell suspension was stained by 10 μM DiO for 30 min at 4°C, re-suspended in a medium, and kept at 4°C before injection. Cells were injected into zebrafish embryos at 2 dpf using a microinjector, with approximately 200 cells/embryo. The larvae with the same amount of fluorescence were randomly divided into different groups and transferred to 12-well culture plates (20 per sample, *n* = 3) at 3 dpf. The groups in this experiment included the normal group, the HepG2 xenograft model group, the positive control group (100 ng ml^−1^ cisplatin), and the drug groups at different concentrations. A total of 10 zebrafish larvae were randomly selected at 6 dpf and photographed in each group using an Axio Zoom V16 fluorescence microscope (Zeiss, Germany), and the relative fluorescence area was calculated. Then, the inhibitory effect of each antitumor drug was quantitatively evaluated (Zhao et al., 2020).

### 2.9 Detection of the zebrafish mRNA expression level by the real-time quantitative polymerase chain reaction

The mRNA expression levels of the key genes were measured using RT-qPCR. The zebrafish HepG2 xenograft model was built. Then, 3 dpf zebrafish embryos were transferred into 12-well plates (20 embryos/well) and incubated with CR-SR (500 μg/ml), CR (300 μg/ml), and SR (1,000 μg/ml) at 72 hpf. The total RNA (1 μg) was converted to first-strand cDNAs using a PrimeScript™ RT reagent Kit for reverse transcription. In 20 μL reactions with each of the forward and reverse primers, cDNA, and SYBR Green Mix, RT-qPCR was performed. Thermal cycling was set at 95°C for 5 s and 60°C for 30 s with 40 cycles. Gene expression for each sample was expressed as a threshold cycle (Ct), normalized to the reference gene *β*-actin (△Ct). The experiment was conducted in triplicate (Lan et al., 2020). Proto-oncogene tyrosine-protein kinase Src (SRC), epidermal growth factor receptor (EGFR), estrogen receptor-α (ESR1), prostaglandin endoperoxide synthase 2 (PTGS2), and amyloid precursor protein (APP) genes were determined, as described previously. The primer sequences of the genes are shown in Table 1 (Carnevali et al., 2010; Lu et al., 2013; Liu et al., 2014; Pashay Ahi et al., 2016; Gu et al., 2020).

**TABLE 1:** Primer sequence used for PCR analysis.

Gene	Forward	Reverse
*EGFR*	5′-ACG​CAG​ACG​AGT​ATT​TAG​TGC​CCA-3′	5′-AGT​TTC​CAA​AGC​TGC​TGT​TCA​GGC-3′
*ESR1*	5′-ACT​GTG​GCT​CGA​TTT​CGG​AGT-3′	5′-TCC​ACT​GGA​CTG​GAG​CAG​AAT​G-3′
*PTGS2*	5′-TGG​ATC​TTT​CCT​GGG​TGA​AGG-3′	5′-GAA​GCT​CAG​GGG​TAG​TGC​AG-3′
*APP*	5′-GGA​GTT​TGT​GTG​CTG​CCC​AA-3′	5′-ACC​GTC​ACC​GTC​TTC​ATC​GT-3′
*SRC*	5′-ACA​CAG​CCC​AAC​ATC​ATC​AA-3′	5′-TAT​CCG​CTC​TCT​CCT​GTC​GT-3′
*β-actin*	5′-TCC​CCT​TGT​TCA​CAA​TAA​CC-3′	5′-TCT​GTT​GGC​TTT​GGG​ATT​C-3′

### 2.10 Statistical analysis

All the assays were carried out in triplicate, and results were expressed as mean values ± standard deviation (mean ± SD). All statistical analyses were carried out by SPSS (Version 25.0) and Origin 2021. The one-way analysis of variance (ANOVA) test was used to check for significant differences among the groups. Differences between models were considered significant when the *p*-value was less than 0.05.

### 2.11 Ethics statement

Zebrafish experiments were conducted according to the Regulation on the Administration of Laboratory Animals (2013 Revision, document number: order no. 638 of the State Council) for experimental care and usage of animals.

## 3 Result and discussion

### 3.1 Chemical profiling

#### 3.1.1 Study on molecular networking of mass spectrometry of CR-SR, CR, and SR decoction

Previously, our laboratory has identified 47 linear diarylheptanoids in CR-SR (Chang et al., 2020). On the basis of this study, we further explored the MS data through the GNPS platform. The MS data were visualized by Cytoscape 3.8.2. The spectral similarities were expressed as the cosine score (cos θ), and the larger the cos θ score, the higher the similarity of the MS/MS fragments (Xue et al., 2021). Also, the structurally similar compounds were inferred by using the differences in secondary mass spectra from related nodes. Each node represented a secondary mass spectral map. A total of 1,120 nodes were incorporated into the MS/MS molecular networking of the CR-SR decoction in the positive mode, resulting in 18 molecular clusters and 809 unconnected nodes (https://gnps.ucsd.edu/ProteoSAFe/status.jsp?task=078b8a6443d540a88720ff67cc3b06a2). Meanwhile, a total of 1,079 nodes were incorporated into the MS/MS molecular networking of the CR decoction in the positive mode, resulting in 22 molecular clusters and 801 unconnected nodes (https://gnps.ucsd.edu/ProteoSAFe/status.jsp?task=87f347a0948e4b92b78cf433b111803a). Based on the clusters in the molecular networking, a total of 12 diarylheptanoids were tentatively identified from CR-SR and CR for the first time in the positive mode.

**FIGURE 2:**
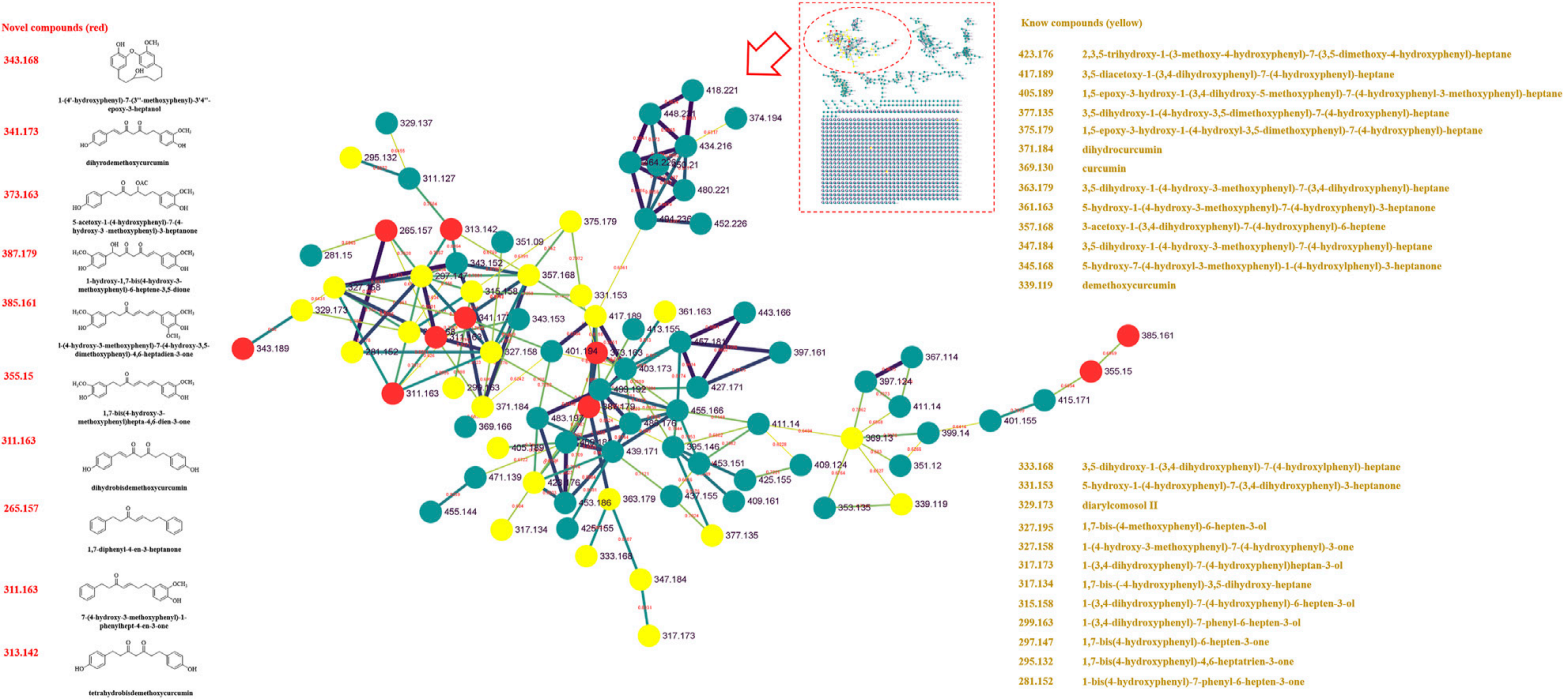
MS/MS molecular networking of the CR-SR decoction in the positive mode. The novel compound is in red, and its name and structure are depicted in the left column. The known compound is in yellow, and the name is depicted in the right column.

**FIGURE 3:**
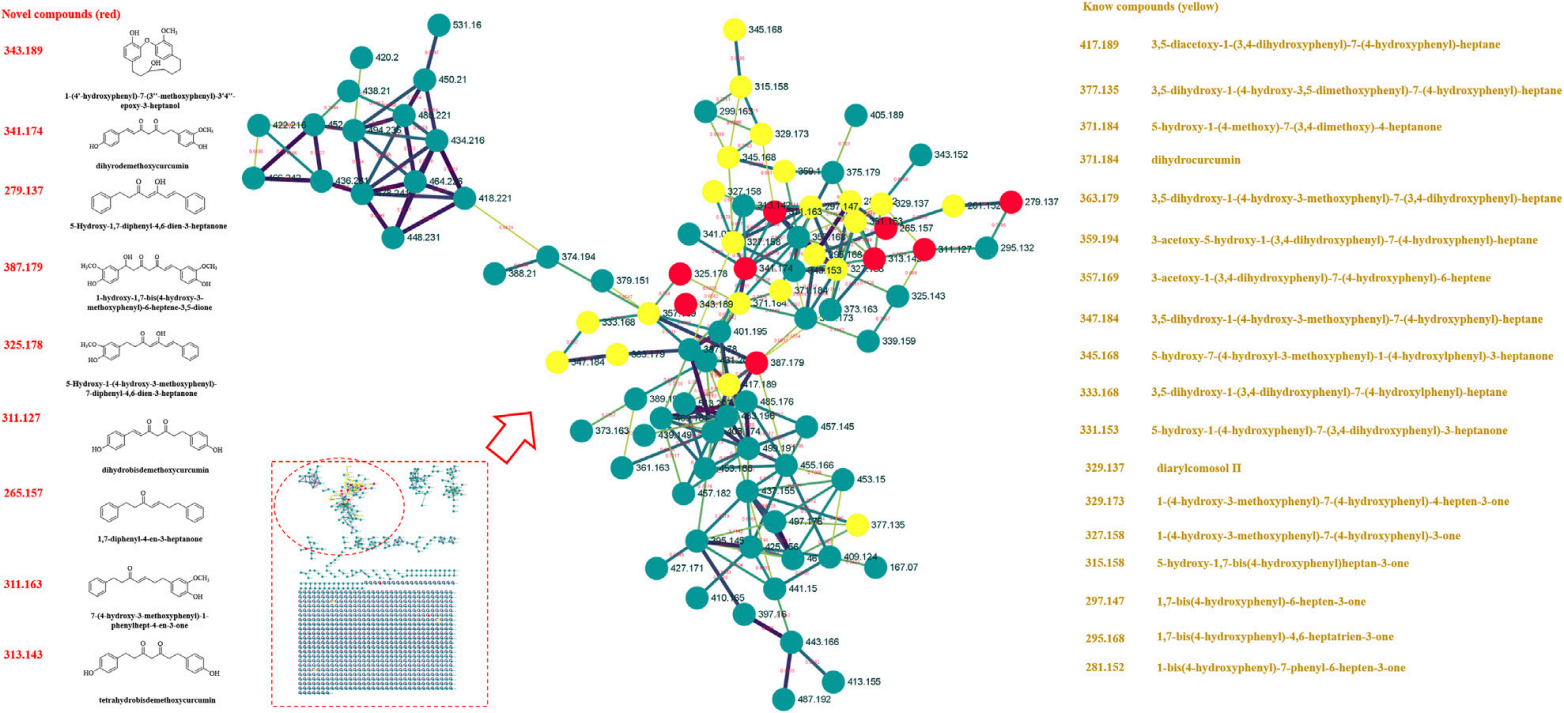
MS/MS molecular networking of the SR decoction in the positive mode. The novel compound is in red, and its name and structure are depicted in the left column. The known compound is in yellow, and the name is depicted in the right column.

In our previous research, diarylheptanoids were the main compounds of CR-SR (Chang et al., 2020). In this molecular map, 25 and 23 diarylheptanoids had been identified in the largest clusters from CR-SR and SR, respectively. Taking *m/z* 311.163 as an example, the compound dissociated into fragment ions [M + H-C_7_H_8_O_2_]^+^ at *m/z* 187, [M + H-C_10_H_12_O]^+^ at *m/z* 163, [M + H-C_7_H_8_O_2_-C_2_H_2_]^+^ at *m/z* 161, [M + H-C_7_H_8_O_2_-C_2_H_4_]^+^ at *m/z* 159, [M + H-C_10_H_12_O_2_]^+^ at *m/z* 147, and [M + H-C_12_H_14_O]^+^ at *m/z* 137 by comparison with the reported literature (Cheng et al., 2018). Its adjacent node at *m/z* 327.158 gave an MS/MS spectrum showing the same characteristic fragments at *m/z* 147. Therefore, the compound was plausibly characterized as 7-(4-hydroxy-3-methoxyphenyl)-1-phenyl-4-hepten-3-one, and its proposed fragmentation pathway is shown in Supplementary Figure S1. According to references, the cracking pathways of other diarylheptanoid compounds could be inferred. Also, other novel diarylheptanoid structures are listed in Table 2, Figure 2, and Figure 3.

**TABLE 2:** Identification of the novel diarylheptanoids of CR-SR and CR decoction by the molecular network.

No.	Name	*t*_R_/min	Empirical formula	Theoretical mass (*m/z*)	Experimental mass (*m/z*)	Mass error (ppm)	MS^2^ data (measured)	CR-SR	CR	SR
**C1**	1,7-Bis(4-hydroxy-3-methoxyphenyl)hepta-4,6-dien-3-one	17.07	C_21_H_23_O_5_	355.15400	355.15265	−3.802	337, 161, 145, and 137	+	−	−
**C2**	5-Acetoxy-1-(4-hydroxyphenyl)-7-(4-hydroxy-3 -methoxyphenyl)-3-heptanone	19.79	C_21_H_25_O_6_	373.16456	373.16287	−4.524	193	+	−	−
**C3**	7-(4-Hydroxy-3-methoxyphenyl)- 1-phenyl-4-hepten-3-one	19.91	C_20_H_23_O_3_	311.16417	311.16275	−4.567	187, 163, 161, 159, 147, and 137	+	+	−
**C4**	5-Hydroxy-1,7-diphenyl-4,6-dien-3-heptanone	20.08	C_19_H_19_O_2_	279.13795	279.13687	−3.892	133, 131, and 261	−	+	−
**C5**	Tetrahydrobisdemethoxycurcumin	20.46	C_19_H_21_O_4_	313.14343	313.14212	−4.201	147 and 107	+	+	−
**C6**	Dihyrodemethoxycurcumin	20.88	C_20_H_21_O_5_	341.13835	341.13681	−4.515	323, 189, 147, 146, and 118	+	+	−
**C7**	1,7-Diphenyl-4-en-3-heptanone	22.60	C_19_H_21_O	265.15869	265.15738	−4.947	247 and 161, 143, and 133	+	+	−
**C8**	1-Hydroxy-1,7-bis(4-hydroxy-3-methoxyphenyl)-6-heptene-3,5-dione	23.01	C_21_H_23_O_7_	387.14383	387.14203	−4.648	369, 199, 194, and 166	+	+	−
**C9**	l-(4-Hydroxy-3-methoxyphenyl)-7-(4-hydroxy-3,5-dimethoxyphenyl)-4,6-heptadien-3-one	23.01	C_22_H_26_O_6_	386.17239	386.12015	−1.549	367, 233, 219, and 175	+	−	−
**C10**	Dihydrobisdemethoxycurcumin	24.73	C_19_H_19_O_4_	311.12778	311.12637	−4.550	225 and 147	+	+	−
**C11**	1-(4′-Hydroxyphenyl)-7-(3″-methoxyphenyl)-3′4″-epoxy-3-heptanol	27.11	C_21_H_27_O_4_	343.19038	343.15271	−3.760	187, 137, and 131	+	+	−
**C12**	5-Hydroxy-1-(4-hydroxy-3-methoxyphenyl)-7-diphenyl-4,6-dien-3-heptanone	28.51	C_20_H_21_O_4_	325.14343	325.14240	−3.185	307, 179, 137, and 131	−	+	−

#### 3.1.2 Identification of the major components by UPLC-LTQ-Orbitrap MS^n^

**FIGURE 4:**
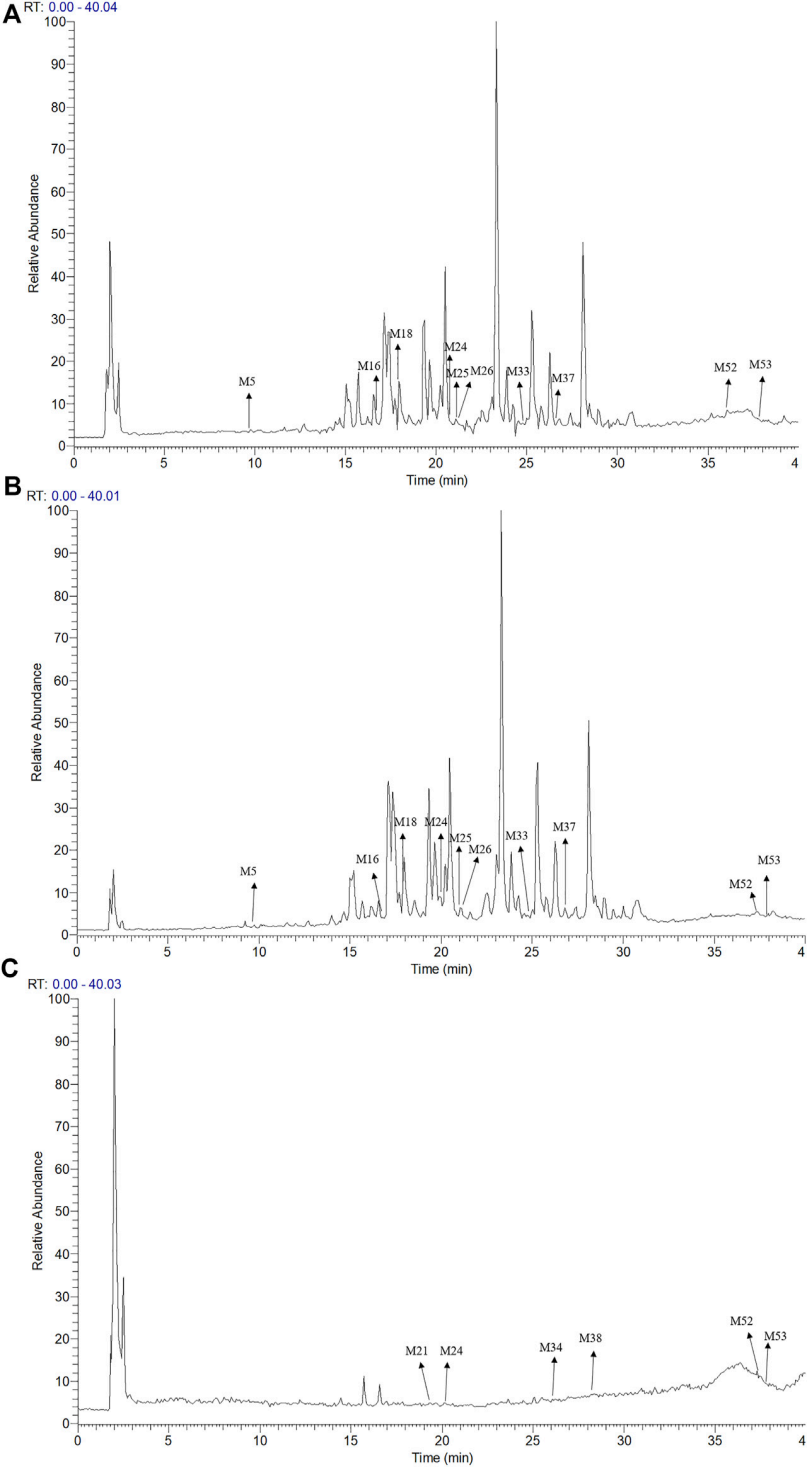
TIC chromatograms in the positive mode; **(A)** CR-SR extract, **(B)** CR extract, and **(C)** SR extract.

**FIGURE 5:**
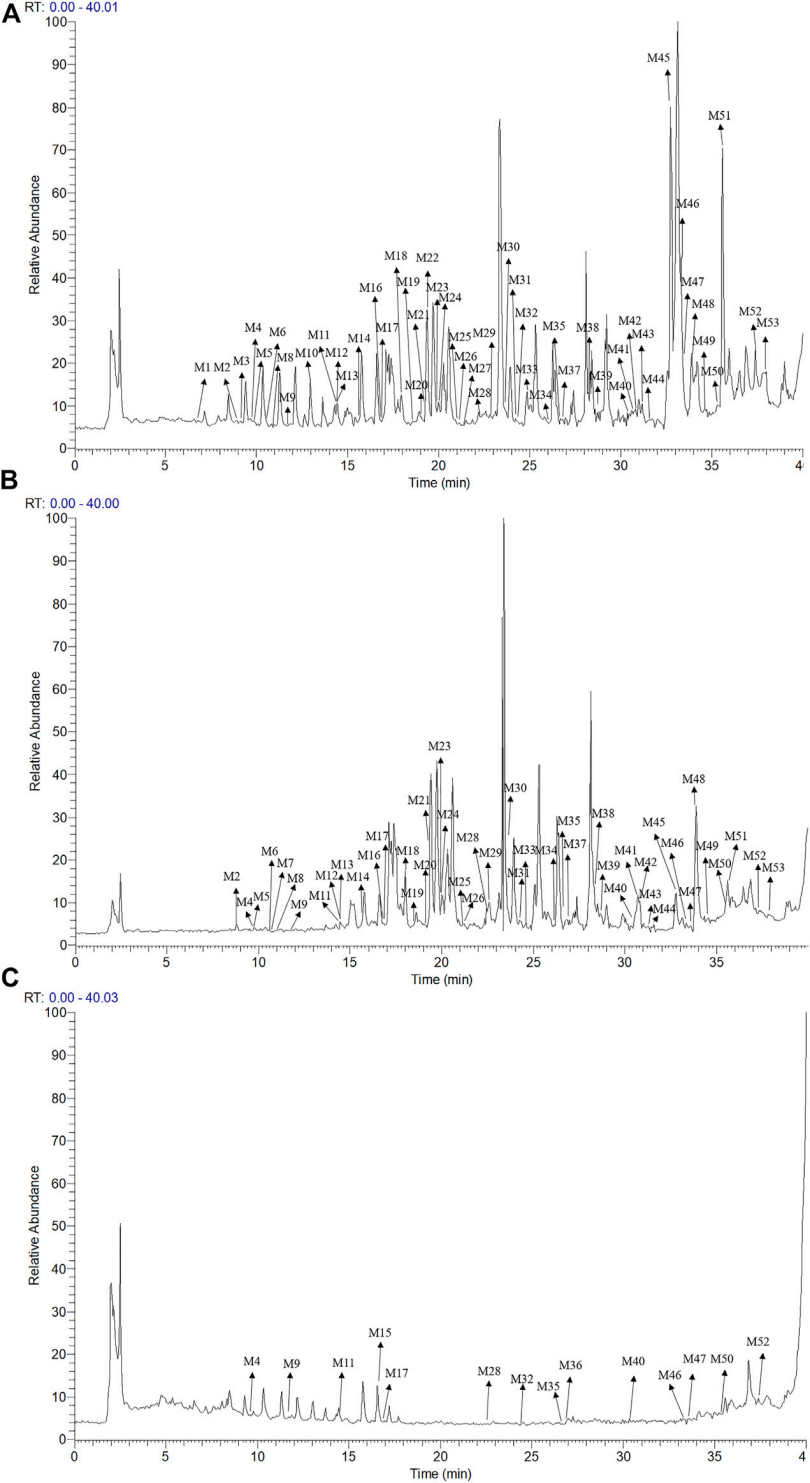
TIC chromatograms in the negative mode; **(A)** CR-SR extract, **(B)** CR extract, and **(C)** SR extract.

**FIGURE 6:**
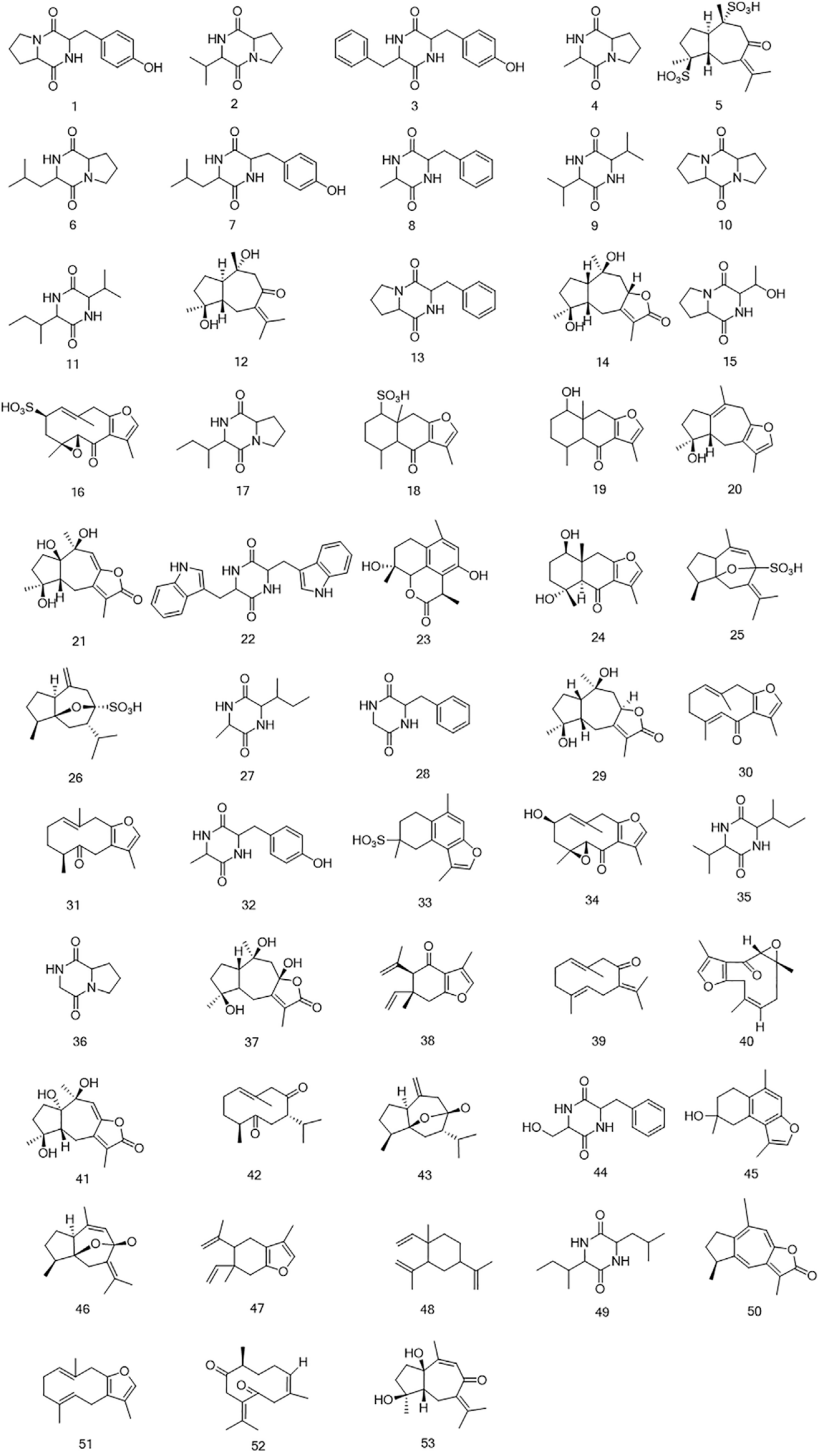
The structures of sesquiterpenoids and cyclic dipeptides identified in CR-SR, CR, and SR extracts.

The UPLC-LTQ-Orbitrap MS^n^ method was used for the comprehensive analysis of sesquiterpenoids and cyclic dipeptide components in CR-SR, CR, and SR. The components were identified by the molecular weight, elemental compositions, retention time (*t*_R_), and characteristic MS/MS fragment mass spectra compared with those reported in the literature. A total of 53 components were preliminarily identified from CR-SR, CR, and SR. A total of 32 sesquiterpenoids (M5, M12, M14, M16, M18–21, M23–26, M29–31, M33–34, M37–43, M45–48, and M50–53) were detected and tentatively characterized from CR-SR and CR in both positive and negative modes. Among them, nine compounds were detected in the SR extract. Cyclic dipeptides are the common cyclopeptides found in nature (Guo et al., 2009a). A total of 21 cyclic dipeptides (M1–4, M6–11, M13, M15, M17, M22, M27–28, M32, M35–36, M44, and M49) were detected and tentatively characterized from CR-SR, CR, and SR extracts in the positive mode. Therein, 18, 13, and 10 cyclic dipeptides were detected from CR-SR, CR, and SR, respectively. The total ion chromatograms (TIC) of CR-SR, CR, and SR in positive and negative ion modes are presented in Figure 4 and Figure 5. The detailed information and structures of the identified components are shown in Table 3 and Figure 6.

**TABLE 3:** Summary of components in CR-SR, CR, and SR extracts.

No.	Name	*t*_R_/min	Empirical formula	Theoretical mass (*m/z*)	Experimental mass (*m/z*)	Adduct	Mass error	MS^2^ data (measured)	CR-SR	CR	SR
							(ppm)				
**M1**	Cyclo(Tyr-Pro)	6.94	C_14_H_17_N_2_O_3_	261.12336	261.12238	M + H	−3.787	261, 233, 216, 215, 188, 136, and 70	+	−	−
**M2**	Cyclo(Val- Pro)	8.87	C_10_H_17_N_2_O_2_	197.12845	197.12758	M + H	−4.435	197, 169, 154, 152, 151, 141, 124, 113, 98, 72, and 70	+	+	−
**M3**	Cyclo(Phe-Tyr)	9.06	C_18_H_19_N_2_O_3_	311.1390	311.13916	M + H	0.452	311, 283, 266, 265, 255, 220, 136, and 120	+	−	−
**M4**	Cyclo(Ala-Pro)	9.70	C_8_H_13_N_2_O_2_	169.09715	169.09633	M + H	−4.974	169, 154, 141, 124, 113, 113, 98, 96, 85, and 70	+	+	+
**M5**	Shogasulfonic acid A	9.75	C_15_H_23_O_7_S_2_	379.08797	379.08624	M-H	−4.566	379, 361, 351,334, 318, 299, 219, and 191	+	+	−
**M6**	Cyclo(Pro-Leu)	10.52	C_11_H_19_N_2_O_2_	211.14410	211.14313	M + H	−4.615	211, 183, 166, 165, 154, 138, 127, 86, 72, and 70	+	+	−
**M7**	Cyclo(Tyr-Leu)	10.71	C_15_H_21_N_2_O_3_	277.15350	277.15466	M + H	−4.218	277, 249, 232, 231, 221, 220, 204, 186, 164, 136, 86, and 72	−	+	−
**M8**	Cyclo(Ala-Phe)	10.98	C_12_H_15_N_2_O_2_	219.11280	219.11206	M + H	−4.720	219, 191, 174, 173, 163, 146, 129, 120, and 72	+	+	−
**M9**	Cyclo(Val-Val)	11.64	C_10_H_19_N_2_O_2_	199.14410	199.14352	M + H	−2.934	199, 171, 156, 154, 153, 143, 126, 108, 100, and 72	+	+	+
**M10**	Cyclo(Pro-Pro)	12.89	C_10_H_15_N_2_O_2_	195.11280	195.11201	M + H	−4.071	195, 167, 150, 149, 139, 122,111, and 70	+	−	−
**M11**	Cyclo(Ile-Val)	14.50	C_11_H_21_N_2_O_2_	213.15975	213.15961	M + H	−0.678	213, 195, 185, 168, 167, 157, 156, 140, 100, 86, 72, and 69	+	+	+
**M12**	Cyclo(Pro-Phe)	14.56	C_14_H_17_N_2_O_2_	245.12845	245.12747	M + H	−4.016	245, 217, 200, 199, 189, 172, 161, 154, 120, 98, and 70	+	+	−
**M13**	Zedoarondiol	14.56	C_15_H_25_O_3_	253.17982	253.17880	M + H	−4.033	253, 235, 225, 217, 207, 189, 175, and 161	+	+	−
**M14**	Zedoalactone C	15.60	C_15_H_23_O_4_	267.15799	267.15908	M + H	−4.101	249, 231, 223, 205, 187, 173, 159, and 145	+	+	−
**M15**	Cyclo(Pro-Thr)	16.51	C_9_H_15_N_2_O_3_	199.10698	199.10771	M + H	−3.771	199, 181, 171, 154, 153, 143, 126, 115, 98, 74, and 70	−	−	+
**M16**	Shogasulfonic acid B	16.81	C_15_H_17_O_6_S	325.07403	325.07431	M-H	0.845	325, 310, 297, 283, 268, 253, 245, 240, 225, and 217	+	+	−
**M17**	Cyclo(Ile-Pro)	16.84	C_11_H_19_N_2_O_2_	211.14410	211.14316	M + H	−4.473	211, 183, 166, 165, 154, 138, 86, 70, and 69	+	+	+
**M18**	Shogasulfonic acid C	18.01	C_15_H_19_O_5_S	311.09503	311.09477	M-H	0.833	311, 293, 283, 247, 231, 203, 189, and 175	+	+	−
**M19**	Dihydrocurcolone	18.58	C_15_H_21_O_3_	249.14852	249.14755	M + H	−3.897	249, 231, 221, 207, 203, 189, 175, 161, and 157	+	+	−
**M20**	Curcumafuranol	19.17	C_15_H_21_O_2_	233.15360	233.15250	M + H	−4.746	233, 215, 197, 191, 187, 173, 159, and 145	+	+	−
**M21**	Zedoalactone B	19.31	C_15_H_21_O_5_	281.13835	281.13828	M + H	−0.250	281, 263, 253, 245, 237, 235, 227, 217, 201, 199, and 183	+	+	−
**M22**	Cyclo(Trp-Trp)	19.42	C_22_H_21_N_4_O_2_	373.16590	373.16422	M + H	−4.509	373, 345, 327, 300, 282, 243, 187, and 159	+	−	−
**M23**	Phacadinane C	20.02	C_15_H_19_O_4_	263.12778	263.12650	M + H	−4.886	263, 245, 235, 227, 219, 217, 203, and 199	+	+	−
**M24**	Curcolonol	20.07	C_15_H_21_O_4_	265.14343	265.14215	M + H	−4.849	265, 247, 249, 229, 219, 209, 201, 191, 173, 145, and 113	+	+	−
**M25**	Shogasulfonic acid D	21.08	C_15_H_21_O_4_S	297.11566	297.11550	M-H	0.517	297, 282, 279, 269, 253, 233, 217, 199, 177, and 149	+	+	−
**M26**	Shogasulfonic acid E	21.16	C_15_H_23_O_4_S	299.13156	299.13115	M-H	1.349	299, 284, 281, 271, 235, 219, 205, and 179	+	+	−
**M27**	Cyclo(Ala-Ile)	21.37	C_9_H_17_N_2_O_2_	185.12845	185.12817	M + H	−1.536	185, 157, 140, 139, 129, 128, 86, 72, and 69	+	−	−
**M28**	Cyclo(Gly-Phe)	22.45	C_11_H_13_N_2_O_2_	205.09715	205.09616	M + H	−4.847	205, 177, 160, 159, 149, 132, 120, 114, and 58	+	+	+
**M29**	Zedoalactone A	22.77	C_15_H_23_O_4_	267.15908	267.15778	M + H	−4.887	267, 249, 239, 231, 223, 221, 207, 203, 189, and 187	+	+	−
**M30**	Furanodienone	23.51	C_15_H_19_O_2_	231.13795	231.13686	M + H	−4.743	231, 216, 213, 203, 189, 187, 185, 173, 161, and 145	+	+	−
**M31**	Furanogermacrene	24.28	C_15_H_21_O_2_	233.1536	233.15256	M + H	−4.448	233, 217, 215, 205, 197, 191, 187, 177, 175, 159, 149, and 145	+	+	−
**M32**	Cyclo(Ala-Tyr)	24.34	C_12_H_15_N_2_O_3_	235.10771	235.10710	M + H	−2.632	235, 207, 190, 189, 179, 162, 144, 136, and 72	+	−	+
**M33**	Furanocadina-1 (10),6,8-triene- 4-sulfonic acid	24.85	C_15_H_17_O_4_S	293.08441	293.08420	M-H	0.695	293, 213, 211, 199, 197, 159, 157, and 145	+	+	−
**M34**	Wenyujin K	26.11	C_15_H_19_O_4_	263.12778	263.12650	M + H	−4.886	263, 245, 235, 227, 217, 203, and 199	+	+	−
**M35**	Cyclo(Ile-Ile)	26.67	C_12_H_23_N_2_O_2_	227.17540	227.17450	M + H	−3.981	227, 199, 181, 171, 170, 154, 136, 86, and 69	+	+	+
**M36**	Cyclo(Gly-Pro)	26.83	C_7_H_11_N_2_O_2_	155.08150	155.08093	M + H	−3.702	155, 127, 110, 109, 82, 71, 70, and 64	−	−	+
**M37**	Zedoarolide B	26.84	C_15_H_21_O_5_	281.13835	281.13846	M-H	0.290	281, 263, 245, 237, 223, 219, 201, 209, and 191	+	+	−
**M38**	Curzerenone	28.31	C_15_H_19_O_2_	231.13795	231.13693	M + H	−4.410	231, 213, 203, 195, 189, 185, 175, 173, 171, and 161	+	+	−
**M39**	Germacrone	28.74	C_15_H_23_O	219.17434	219.17340	M + H	−4.297	219, 201, 191, 177, 163, 159, 149, 145, 137, 131, 123, and 109	+	+	−
**M40**	Zederone	30.46	C_15_H_19_O_3_	247.13287	247.13171	M + H	−4.698	247, 229, 219, 211, 205, 201, 187, and 183	+	+	+
**M41**	Zedoalactone D	30.66	C_15_H_21_O_5_	281.13835	281.13745	M + H	−3.020	281, 263, 253, 245, 217, 203, 189, 171, 163, and 149	+	+	−
**M42**	Curdione	30.79	C_15_H_25_O_2_	237.18490	237.18375	M + H	−4.876	237, 219, 201, 191, 177, 159, 149, 135, 133, 121, and 107	+	+	−
**M43**	Curcumol	31.21	C_15_H_25_O_2_	237.18490	237.1875	M + H	−4.876	237, 223, 219, 208, 194, 190, 176, and 162	+	+	−
**M44**	Cyclo(Phe-Ser)	31.54	C_12_H_15_N_2_O_3_	235.10884	235.10770	M + H	4.769	235, 217, 207, 190, 189, 179, 162, 147, 120, 113, and 60	+	+	−
**M45**	Furanocadina-1 (10),6,8-triene-4-ol	32.80	C_15_H_19_O_2_	231.13696	231.13795	M + H	−4.311	231, 217, 213, 203, 189, 185, 177, 171, 163, 159, 149, and 145	+	+	−
**M46**	Curcumenol	33.30	C_15_H_23_O_2_	235.16925	235.16829	M + H	−4.110	235, 217, 207, 199, 189, 179, 177, 175, 161, 149, and 147	+	+	+
**M47**	Curzerene	33.56	C_15_H_21_O	217.15869	217.15776	M + H	−4.291	217, 199, 189, 175, 171,161, 159, 149, 145, and 123	+	+	+
**M48**	β-Elememe	33.99	C_15_H_25_	205.19415	205.19507	M + H	−4.515	205, 190, 177, 165, 163, 149, 135, 123, and 121	+	+	−
**M49**	Cyclo(Leu-Ile)	34.56	C_12_H_23_N_2_O_2_	227.17540	227.17491	M + H	−2.771	227, 199, 181, 171, 154, 136, 114,86, 72, and 69	+	+	−
**M50**	Gweicurculactone	35.46	C_15_H_17_O_2_	229.12230	229.12132	M + H	−4.350	229, 214, 211, 201, 185, 173, 159, and 143	+	+	+
**M51**	Furanodiene	35.59	C_15_H_21_O	217.15869	217.15758	M + H	−1.112	217, 199, 189, 175, 161, 157, 147, and 133	+	+	-
**M52**	Dehydrocurdione	37.38	C_15_H_23_O_2_	235.16925	235.16824	M + H	−4.322	235, 217, 207, 199, 189, 175, 161, and 147	+	+	+
**M53**	Aerugidiol	37.79	C_15_H_21_O_3_	249.14852	249.14891	M-H	1.5610	249, 231, 221, 216, 209, 193, 181, and 165	+	+	+

The sesquiterpenoids were more easily detected in the positive mode. The six sulfonated sesquiterpenoids were only shown in the negative mode. The 32 identified sesquiterpenoids were divided into six categories. The main kind of sesquiterpenoid was the gaiane-type, including compounds M5, M12, M14, M20, M21, M25, M26, M29, M37, M41, M43, M46, M50, and M53. Compounds M16, M30, M31, M34, M39, M40, M42, and M51 were germacrane-type sesquiterpenoids. Compounds M38, M47, and M48 were elemane-type sesquiterpenoids, and compounds M18, M19, and M24 were eudesmane-type sesquiterpenoids. In addition, the cadiane-type sesquiterpenoid M23 and marathon-type sesquiterpenoids M33 and M35 were identified.

Cyclic dipeptides have the same skeleton, which differs only in the side-chain substituent at C (3) and C (6) of the cyclic dipeptide ring. The product ions [M + H-CO]^+^, [M + H-HCONH_2_]^+^, [M + H-CO-HCONH_2_]^+^, and [M + H-CO-CO]^+^ were associated with the cyclic dipeptide skeleton (Furtado et al., 2007). The cleavage pathway of the cyclic dipeptide skeleton is shown in Supplementary Figure S2. A series of low-mass ions associated with amino acid residues and fragmentation patterns [M + H-R]^+^ were formed by the *i*-cleavage of amino acid residues. The ions [M + H-CO-CO-R]^+^ could be considered as diagnostic for substituents at C (3) and C (6). The ions at *m/z* 60 (C_2_H_6_NO, Ser), *m/z* 70 (C_4_H_8_N, Pro), *m/z* 72 (C_4_H_10_N, Val), *m/z* 74 (C_3_H_8_NO, Thr), *m/z* 120 (C_8_H_10_N, Phe), *m/z* 129 (C_5_H_9_N_2_O_2_, Ala), *m/z* 136 (C_8_H_10_NO, Tyr), and *m/z* 159 (C_10_H_11_N_2_, Trp) were characterized as the specific amino acid residues. The ions at *m/z* 86 (C_8_H_10_N) and 69 (C_5_H_9_) were indicative of the amino acid residue Ile, while the ions at *m/z* 86 (C_8_H_10_N) and *m/z* 72 (C_3_H_6_NO) indicated the presence of the amino acid residue Leu (Xing et al., 2008; Guo et al., 2009b). Furthermore, the fragment ions at *m/z* [M + H-C_2_H_4_]^+^ and [M + H-C_2_H_4_-2CO-R]^+^ were produced by Pro. Cyclic dipeptides contained a Ser- or Thr-generated fragment ion at *m/z* [M + H-H_2_O]^+^. The formation of each amino acid residue is shown in Supplementary Figure S2.

### 3.2 Network analysis and molecular docking

#### 3.2.1 Target prediction

**FIGURE 7:**
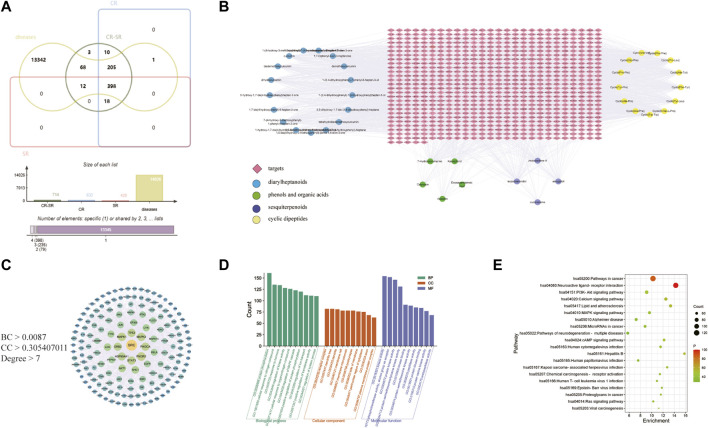
Network pharmacology of CR-SR in liver cancer treatment. **(A)** Venn diagram of active compound targets of CR-SR, CR, SR with liver cancer targets. **(B)** Active compound-target network of the overlapping targets between CR-SR and liver cancer (targets as orange diamond nodes; diarylheptanoids as blue circle nodes; phenols and organic acids as green circle nodes; sesquiterpenoids as purple circle nodes; cyclic dipeptides as yellow circle nodes. **(C)** The PPI network of the screening targets. **(D)** Go enrichment analysis (top 10). **(E)** Bubble diagram of enrichment analysis of KEGG pathway (top 20).

The identified components were used as a chemical information database for network analysis research. After searching for targets, 714, 632, and 428 compound targets in CR-SR, CR, and SR were selected for network analysis, respectively. “Liver cancer” was the keyword used searching for disease-related targets, and 14,026 liver cancer-associated targets were retrieved. Then, 683, 604, and 410 overlapping targets were selected as the potential targets for CR-SR, CR, and SR against liver cancer by the Venn tool (Figure 7A).

Then, 684, 604, and 410 overlapping targets were selected as the potential targets for CR-SR, CR, and SR against HCC by the Venn tool.

#### 3.2.2 Network construction and analysis

The active compounds and potential targets were imported into Cytoscape 3.8.2 software to visualize the active compound–target network (Chen et al., 2019). In Figure 7B, there were a total of 39 active compounds in CR-SR associated with these potential targets, which consisted of 746 target nodes in the network. Apparently, the same targets could correspond to multiple active compounds, and different targets might also correspond to the same active compounds. The complex characteristics of multi-compound and multi-target effects in the network could be easily detected.

The potential targets were input into the STRING database to construct the PPI network. In this network, there were three main parameters, namely, “degree,” “betweenness,” and “closeness,” which were used as filters to select the key targets. The plug-in CytoNCA was used to extract nodes with higher scores in the network, taking the median of betweenness, closeness, and degrees of freedom as the cut point. The targets above the cut point were considered critical targets (Bao et al., 2020). As shown in Figure 7C, the screened PPI network consisted of 162 nodes and 1,652 edges in CR-SR. These key targets contained SRC, TP53, MAPK1, PIK3CA, EGFR, and ESR1, among others. It is speculated that these targets have an important role in the treatment of liver cancer by CR-SR, which is worthy of further exploration.

#### 3.2.3 Gene function and pathway enrichment

**FIGURE 8:**
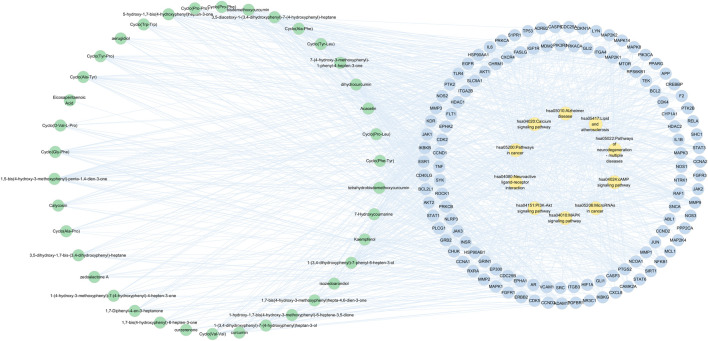
Active compound-key target-pathway network of CR-SR for liver cancer, where green nodes resented active compounds, blue nodes denoted key targets, and orange nodes indicated enriched pathway.

GO annotation analysis and KEGG pathway analysis were conducted on the key targets by using the Metascape platform (Yu et al., 2021). The results are shown in Figure 7. The top 10 terms in the biological process (BP), cellular component (CC), and molecular function (MF) are displayed in Figure 7D. BP-related items mainly involved protein phosphorylation, positive regulation of protein phosphorylation, and cellular response to nitrogen compounds. As for CC-related items, potential targets were mainly concentrated in the membrane raft, membrane microdomain, and receptor complex. MF-related items involved kinase activity, protein kinase activity, and phosphotransferase activity. The top 20 pathways of the KEGG enrichment analysis are shown in Figure 7E. KEGG enrichment analysis showed that the main pathways significantly affected were cancer pathways, the PI3K-Akt signaling pathway, and human papillomavirus infection. To further reveal the molecular mechanism of CR-SR anti-liver cancer compatibility, an “active compound–key target pathway” network was constructed. As shown in Figure 8, the networks were visualized by Cytoscape based on the signaling pathways and targets involved.

#### 3.2.4 Molecular docking

Molecular docking was used to investigate the possibility of interactions between active compounds and key targets (Chen et al., 2019). Then, according to the results of network analysis and the literature, SRC, EGFR, ESR1, PTGS2, and APP were taken as examples (Liu et al., 2020; Yan et al., 2011; Markosyan et al., 2019; Ren et al., 2016; Di Stadio et al., 2017). Five key targets were used for molecular docking (the key docking parameters are displayed in Supplementary Table S1). Generally, the higher the score, the more stable the conformation is. Since the PDB structure of the target APP does not have a proligand, no in-depth analysis is performed. Prior to docking, the DNA chain and water molecules were removed, and the docking receptor was defined by the original ligand. Also, all ligand compounds were carried out through energy minimization (Liu et al., 2021). Most active compounds had stronger interactions with the key targets than those with the original ligand.

**FIGURE 9:**
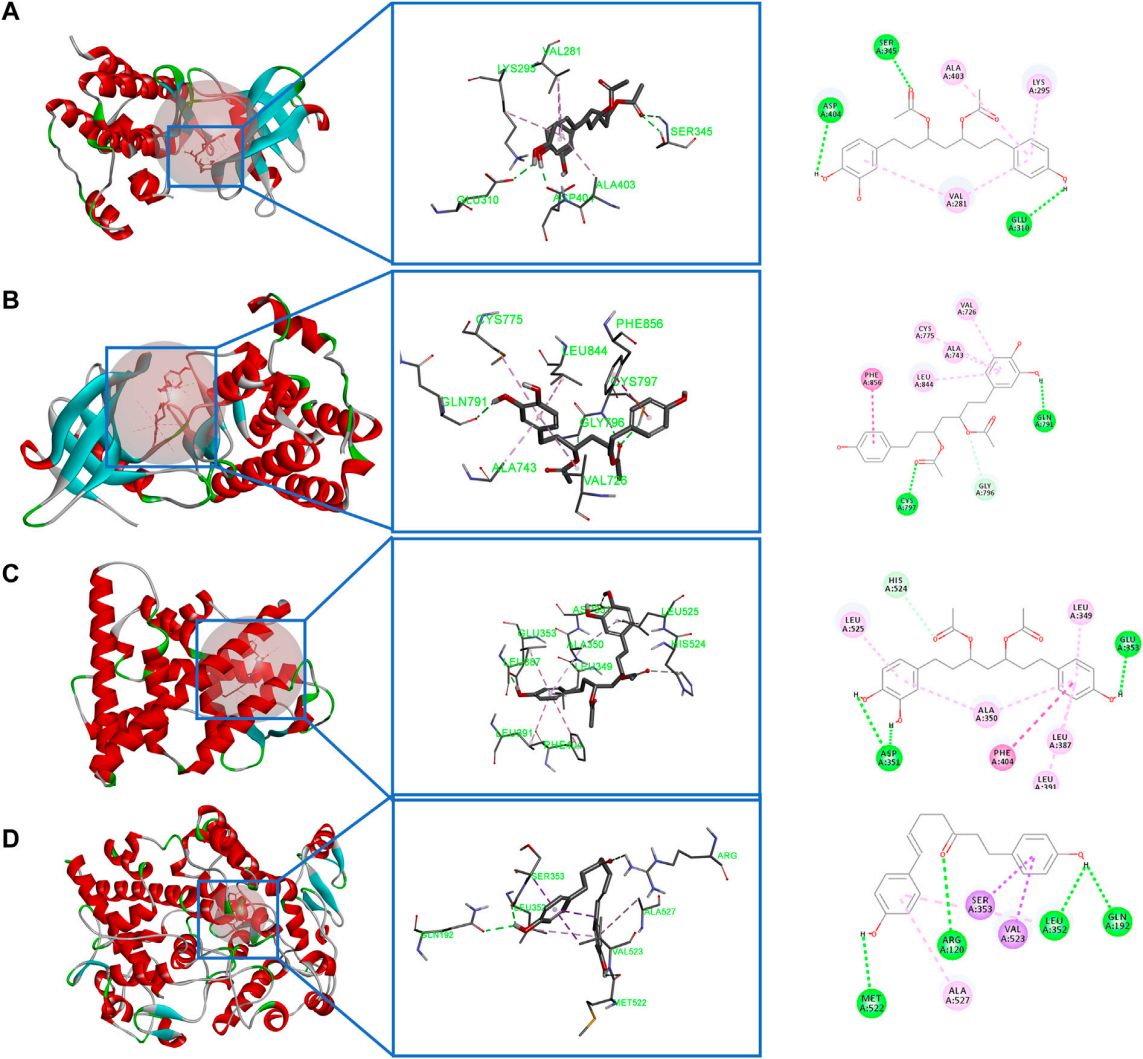
Molecular docking. **(A)** Binding modes of 3,5-diacetoxy-1-(3,4-dihydroxyphenyl)-7-(4-hydroxyphenyl)-heptane in the SRC-binding pocket. **(B)** Binding modes of 3,5-diacetoxy-1-(3,4-dihydroxyphenyl)-7-(4-hydroxyphenyl)-heptane in the EGFR-binding pocket. **(C)** Binding modes of 3,5-diacetoxy-1-(3,4-dihydroxyphenyl)-7-(4-hydroxyphenyl)-heptane in the ESR1-binding pocket. **(D)** Binding modes of 1,7-bis(4-hydroxy-3-methoxyphenyl)hepta-4,6-dien-3-one in the PTGS2-binding pocket.

The results of molecular docking showed that diarylheptanoids and cyclic dipeptides had good binding abilities to corresponding proteins. Interestingly, SRC, EGFR, and ESR1 all showed strong affinity abilities with 3,5-diacetoxy-1-(3,4-dihydroxyphenyl)-7-(4-hydroxyphenyl)-heptane, and PTGS2 showed a good activity for docking with 1,7-bis(4-hydroxy-3-methoxyphenyl)hepta-4,6-dien-3-one. In addition, cyclo(Phe-Tyr) and cyclo(Trp-Trp) in cyclic dipeptide and eicosapentaenoic acid in organic acid showed strong affinity abilities with the aforementioned target. The binding modes of some active compounds docked with EGFR, ESR1, PTGS2, and SRC are shown in Figure 9. Taking 3,5-diacetoxy-1-(3,4-dihydroxyphenyl)-7-(4-hydroxyphenyl)-heptane and SRC as examples, the complex compounds were stabilized by the amino acid residues SER, ASP, and GLU by hydrogen bonds and also bound to ALA, LYS, and VAL by hydrophobic bonds. These docking results indicated the active compounds of CR-SR, CR, and SR had a high binding affinity for key targets of liver cancer.

### 3.3 Cell proliferation assay

**FIGURE 10:**
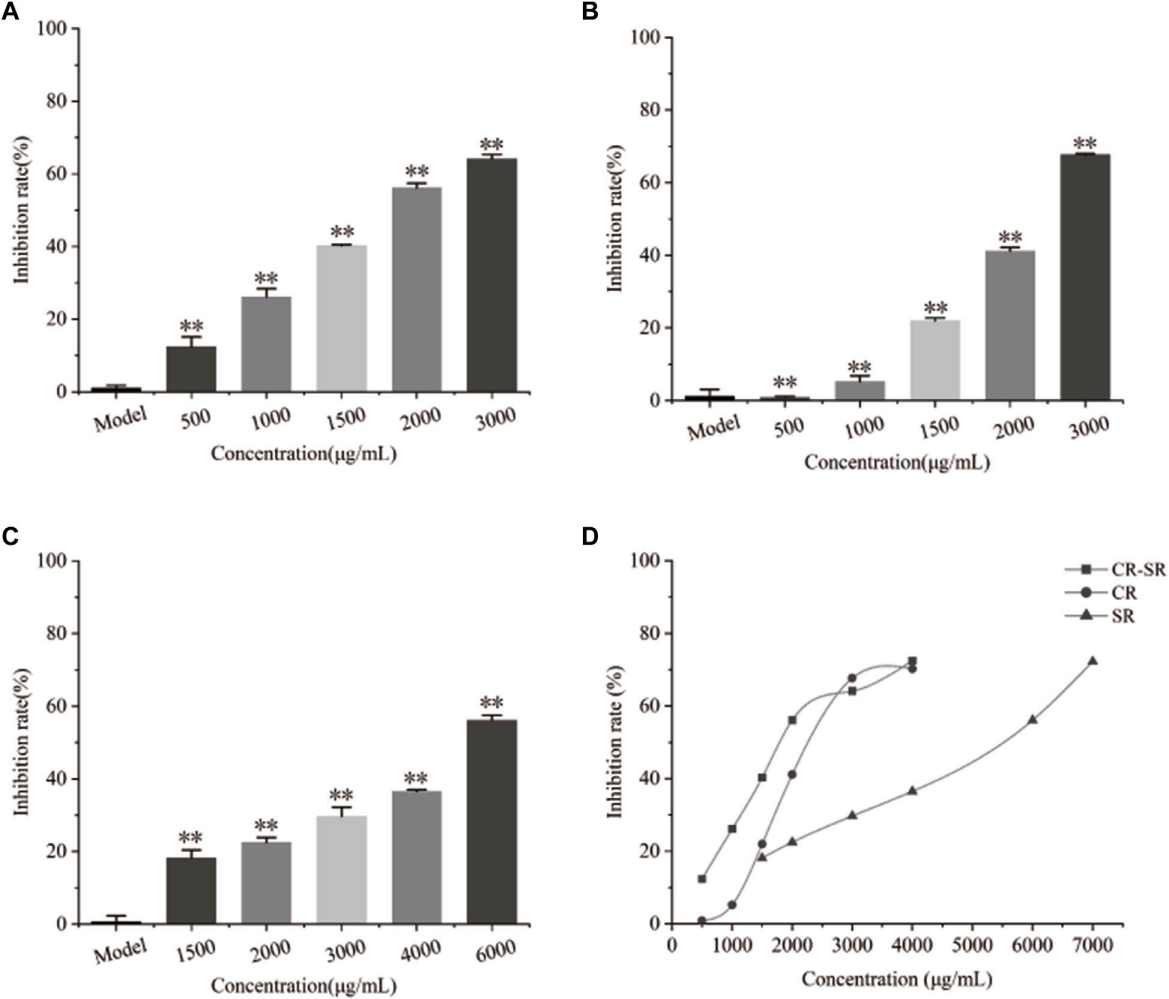
Inhibitory effects on the proliferation rate of HepG2 cells. **p < 0.01 compared with the model group. **(A)** Chart of CR-SR, **(B)** chart of CR, **(C)** chart of SR, and **(D)** line chart of CR-SR, CR, and SR.

As shown in Figure 10, CR-SR, CR, and SR significantly inhibited the proliferation of HepG2 cells after 48 h. As the concentration of the sample increased, cell proliferation rates gradually decreased. Compared with the model group, the treatment groups showed significantly lower cell proliferation rates (*p* < 0.01) (Chen et al., 2018). CR-SR showed significant anti-liver cancer activities than CR and SR (Figure 10D). The IC_50_ values were 1,535, 1,874, and 5,436 μg/ml, respectively. In addition, the SR had the lowest anti-liver cancer activity.

### 3.4 Anti-liver cancer activities of CR-SR, CR, and SR in the zebrafish HepG2 xenograft model

**FIGURE 11:**
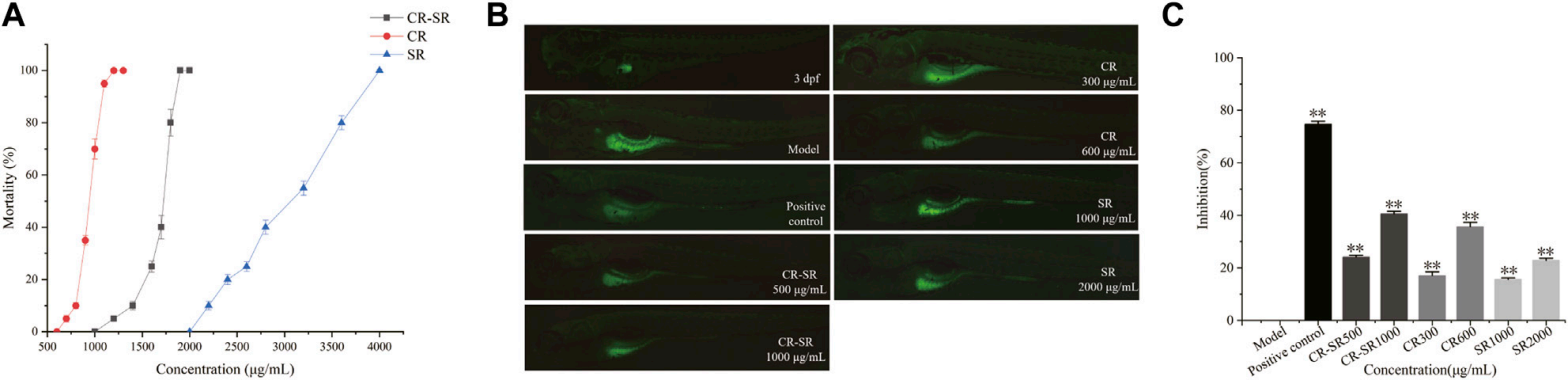
Activity of anti-liver cancer in the zebrafish HepG2 xenograft model. **(A)** Dose–toxicity curves in 3–6 dpf zebrafish. **(B)** Effects of CR-SR, CR, and SR on the tumor area in zebrafish (×40). **(C)** Inhibition rates of the CR-SR, CR, and SR groups. **p < 0.01 compared with the model group.

As shown in Figure 11, the maximum tolerance concentrations (MTCs) of CR-SR, CR, and SR were 1,900, 1,200, and 4,000 μg/ml, respectively (Figure 11A). This indicates that the toxicity was ranked as CR > CR-SR > SR. It indicated that SR had the effect of reducing toxicity. The zebrafish HepG2 xenograft model was used to assess the anti-liver cancer activities of CR-SR and CR. Compared to the model group, the growth of the HepG2 cells was significantly inhibited in the drug treatment group.

At the end of the experiment, the tumor area in the model group was the largest, and the fluorescence was bright and clear. Compared to the model group, the tumor area in each treatment group was reduced, and the fluorescence intensity was weakened (Figure 11B). In addition, the inhibition rates of CR-SR were 40.53% at 1,000 μg/ml and 24.11% at 500 μg/ml. The inhibition rates of CR were 35.66% at 600 μg/ml and 17.44% at 300 μg/ml. The inhibition rates of SR were 22.87% at 2,000 μg/ml and 15.68% at 1,000 μg/ml, respectively. As a positive control, the inhibition rate of cisplatin was 74.72% at 0.1 μg/ml (Figure 11C).

Taken together, the results showed that CR-SR, CR, and SR all had anti-liver cancer effects *in vivo*, which were consistent with those of the cell-level *in vitro* results (Xu et al., 2015). The inhibition of tumor growth of CR-SR is remarkably better than CR and SR at the concentrations of LC_0_ and LC_0_/2, which indicated the inhibitory effect on tumor growth was better than that of single drug pairs (Zhao et al., 2020).

### 3.5 Effects of CR-SR, CR, and SR on the liver cancer-related mRNA expression level in zebrafish larvae

**FIGURE 12:**
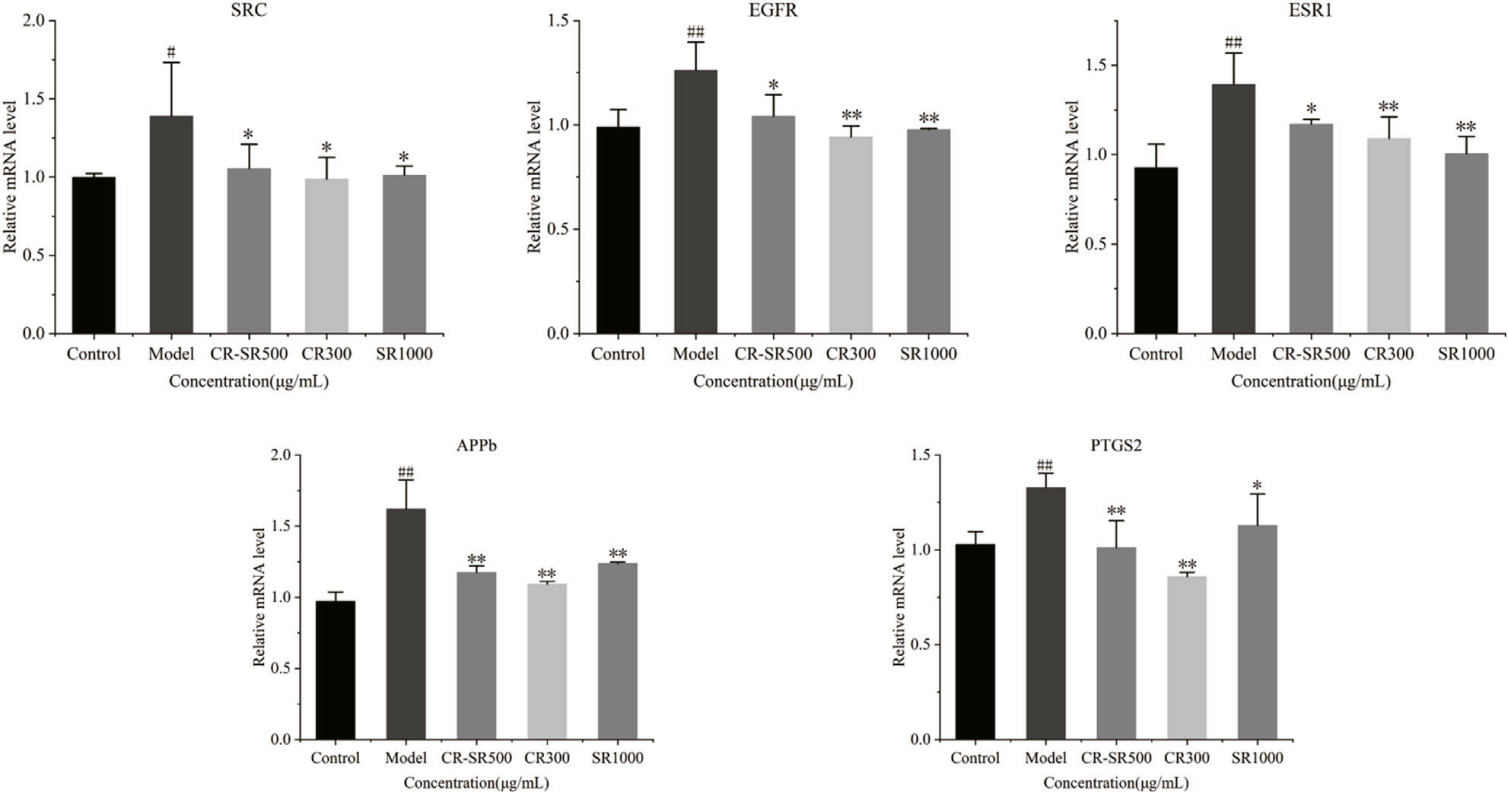
Expression of SRC, EGFR, ESR1, PTGS2, and APP in zebrafish larvae was detected by RT-qPCR.

To clarify the mechanism of anti-liver cancer activities of CR-SR, CR, and SR, the mRNA expression levels were measured for SRC, EGFR, ESR1, PTGS2, and APP. As shown in Figure 12, compared to the control group, the expression levels of SRC, EGFR, ESR1, PTGS2, and APP were apparently upregulated in the model group (*p* < 0.01 or 0.05), while CR-SR, CR, and SR treatment markedly reversed these key targets. These results demonstrated that CR-SR, CR, and SR might exert an anti-liver cancer effect by downregulating the expression of SRC, EGFR, ESR1, PTGS2, and APP.

## 4 Discussion

Based on the previous research, 12 new diarylheptanoids were rapidly identified by the similarity of MS/MS fragments in the molecular network (Chang et al., 2020). A total of 53 compounds were tentatively identified by comparing the retention times and MS data of the detected peaks with those reported in the literature, including 32 sesquiterpenoids and 21 cyclic dipeptides. Specifically, 32 sesquiterpenoids and 18 cyclic dipeptides were deduced in CR-SR, and 32 sesquiterpenoids and 13 cyclic dipeptides were inferred in CR. Nine sesquiterpenoids and 10 cyclodipeptide compounds were derived from SR. The results showed that the main source of sesquiterpenoids was from CR, and the cyclic dipeptides were mainly distributed in both CR and SR, indicating that the types of chemical components in CR and SR were similar. On account of there being no fragmentation rules for sesquiterpenoids, the probable fragmentations of a few sesquiterpenoids were proposed based on the reference literature. In addition, fragmentation pathways of cyclic dipeptides were concluded, which provided a reference for the identification of these compounds.

Our previous research showed CR-SR oil had more remarkable anti-cancer activities than CR or SR (Xu et al., 2015). In this study, the anti-liver cancer activities of CR-SR, CR, and SR were evaluated using the HepG2 cell model *in vitro* and the zebrafish xenograft model *in vivo*. The result indicated that CR-SR, CR, and SR possessed anti-liver cancer activities, and the activities were ranked as CR-SR > CR > SR, which suggested that their compatibility could enhance the effect on the anti-liver cancer activity. These results were consistent with those of previous studies, which might also justify the rationality of botanical drug pair compatibility (Xu et al., 2015). According to the comparison and analysis of CR-SR, CR, and SR from the chemical profile and pharmacological activities, we found that CR contributes more to the anti-liver cancer activity and SR played an important role in reducing acute toxicity.

Network analysis showed that the mechanism for the anti-liver cancer activities of CR-SR, CR, and SR was multi-component, multi-target, and multi-pathway. The results indicated that the key targets of the pathway in cancer were related to the therapeutic effect of anti-liver cancer activities, including SRC, EGFR, ESR1, PTGS2, and APP (Yan et al., 2011; Ren et al., 2016; Di Stadio et al., 2017; Markosyan et al., 2019; Hunter et al., 2020; Yuan et al., 2021). Specifically, SRC was the non-receptor tyrosine kinase. Under physiological status, the SRC physiological status maintained the foundation of cells, which involved in cell survival and proliferation, while in liver cancer, it significantly interferes with the basic activities of cells. For instance, researchers found that the expression of t-Src in liver cancer tissue is significantly higher than that of non-tumor tissue and positively correlated with the tumor stage and cellular differentiation (Ren et al., 2016; Hunter et al., 2020). EGFR was a transmembrane receptor and was related to the proliferation, angiogenesis, and apoptosis of tumor cells (Yuan et al., 2021). ESR1 in the liver was associated with stimulation of hepatocyte proliferation and was highly sensitive to liver fibrosis or hepatic fibrosis (Yan et al., 2011). PTGS2 plays a key role in antitumor immune suppression, and its immune suppressive functions lead to tumor immune evasion and poor survival (Markosyan et al., 2019). APP is a cell membrane protein that is closely related to tumor growth and metastasis and functions as a tumor-promoting factor in cancer (Di Stadio et al., 2017).

The targets in the aforementioned cancer pathways (SRC, EGFR, ESR1, PTGS2, and APP) were used for molecular docking analysis with all active compounds. The docking results showed that diarylheptanoids and cyclic dipeptides had a good binding ability to the corresponding proteins, which preliminarily verified the accuracy of the network analysis results. Specifically, our research indicated that curcumin and demethoxycurcumin exert inhibitory effects by regulating the EGFR target, and similar results have been reported in the literature (Chen and Xu, 2005; Hatamipour et al., 2018). For example, demethoxycurcumin effectively downregulated heat shock protein 70 and maintained the EGFR function *via* activating the AMPK pathway (Hatamipour et al., 2018). Furthermore, our results revealed the anti-liver cancer activities of CR-SR, CR, and SR were associated with the PI3K-Akt signaling pathway and human papillomavirus infection. The PI3K/Akt pathway plays a vital role in many ways in cancer, such as cell growth, survival, and apoptosis survival. Activated Akt could catalyze the phosphorylation of a series of proteins, promote tumor cell growth and proliferation, inhibit apoptosis, enhance invasion and metastasis, and act as a central information substance in the process of tumor cell growth (Chen et al., 2017; Feng et al., 2018).

Network analysis techniques rely on predicting targets from existing databases. The content of each compound in TCM was different, so its content was not considered when using this technology for prediction (Huang et al., 2020). Molecular docking was based on the geometric matching algorithm and lowest energy binding mode search. The downside of molecular docking was that it could only predict the possibility of the drug binding to the target. Both had certain limitations (Fornander et al., 2012). The RT-qPCR technology could analyze and verify the expression patterns of related protein-coding genes and improve the precision and accuracy of results. The expressions of SRC, EGFR, ESR1, PTGS2, and APP were detected by RT-qPCR. The results showed that the expressions of the aforementioned targets were downregulated in CR-SR, CR, and SR, which suggested that CR-SR, CR, and SR can exert an anti-liver cancer effect through the inhibition of SRC, SRC, EGFR, ESR1, PTGS2, and APP. The results indicated that our network analysis-based prediction model of molecular mechanisms was reliable to some extent.

In summary, we systematically analyzed the chemical profiles of CR-SR, CR, and SR. Network analysis, molecular docking, and experimental *in vivo* analysis were used to study the active compounds of the drug pair and the single botanical drugs and to elucidate the mechanisms of CR-SR, CR, and SR against liver cancer. The current method still has room to improve. Apart from computational methods and experimental verification, we can take the dose dependence factor into the network analysis to improve the feasibility and accuracy in future research. The results provided theoretical support for understanding the active compounds and the pharmacological mechanisms of CR-SR, CR, and SR.

## Data Availability

The datasets presented in this study can be found in online repositories. The names of the repository/repositories and accession number(s) can be found in the article/Supplementary Material.

## Abbreviations

CR-SR, *Curcumae Rhizoma* and *Sparganii Rhizoma*; CR, *Curcumae Rhizoma*; SR, *Sparganii Rhizoma*; RT-qPCR, real-time quantitative polymerase chain reaction; TCM, traditional Chinese medicine; GNPS, Global Natural Products Social Networking; MNLC, maximum non-lethal concentration; dpf, days postfertilization; BP, biological process; CC, cellular component; MF, molecular function; SRC, proto-oncogene tyrosine-protein kinase Src; EGFR, epidermal growth factor receptor; ESR1, estrogen receptor-α; PTGS2, prostaglandin endoperoxide synthase 2; APP, amyloid precursor protein; MTC, maximum tolerance concentration.
