# Supplementary material for: Curcumae Rhizoma - combined with Sparganii Rhizoma in the treatment of liver cancer: Chemical analysis using UPLC-LTQ-Orbitrap MSn, network analysis, and experimental assessment
Source: Front Pharmacol. 2022 Dec 5;13:1027687. doi: 10.3389/fphar.2022.1027687 (PMC9764015; doi:10.3389/fphar.2022.1027687)
Supplement: Supplementary file 1 [file DataSheet1.docx]

Supplementary Material

# Supplementary Figures

## Supplementary Figure 1


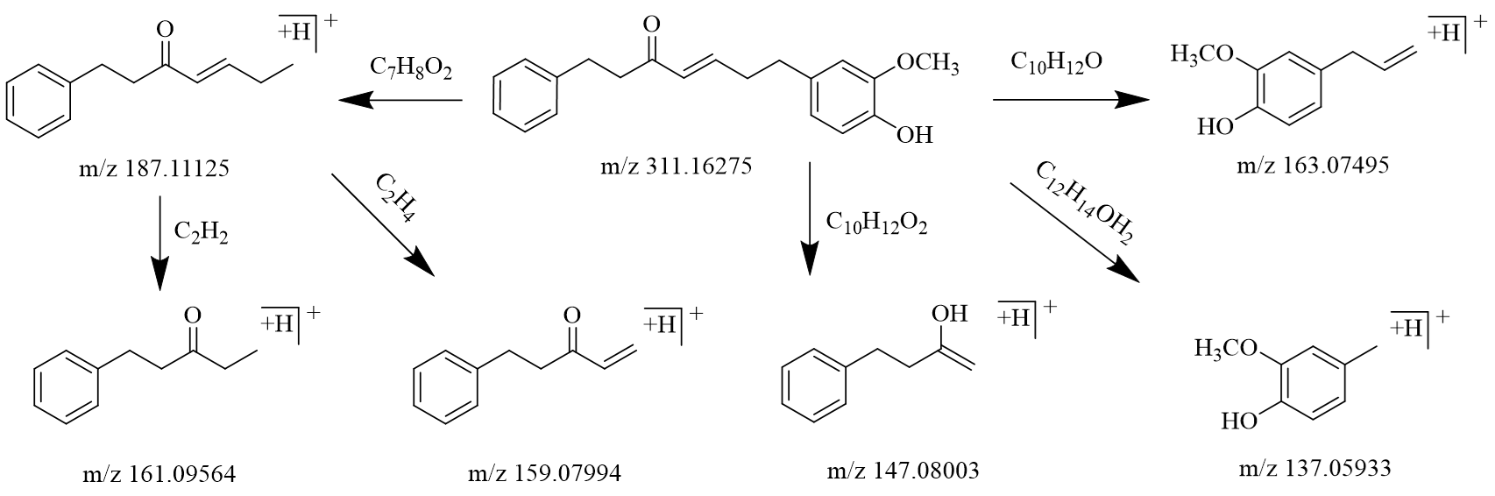


**Supplementary Figure 1**. The proposed fragmentation of compound C3.

## Supplementary Figure 2

#
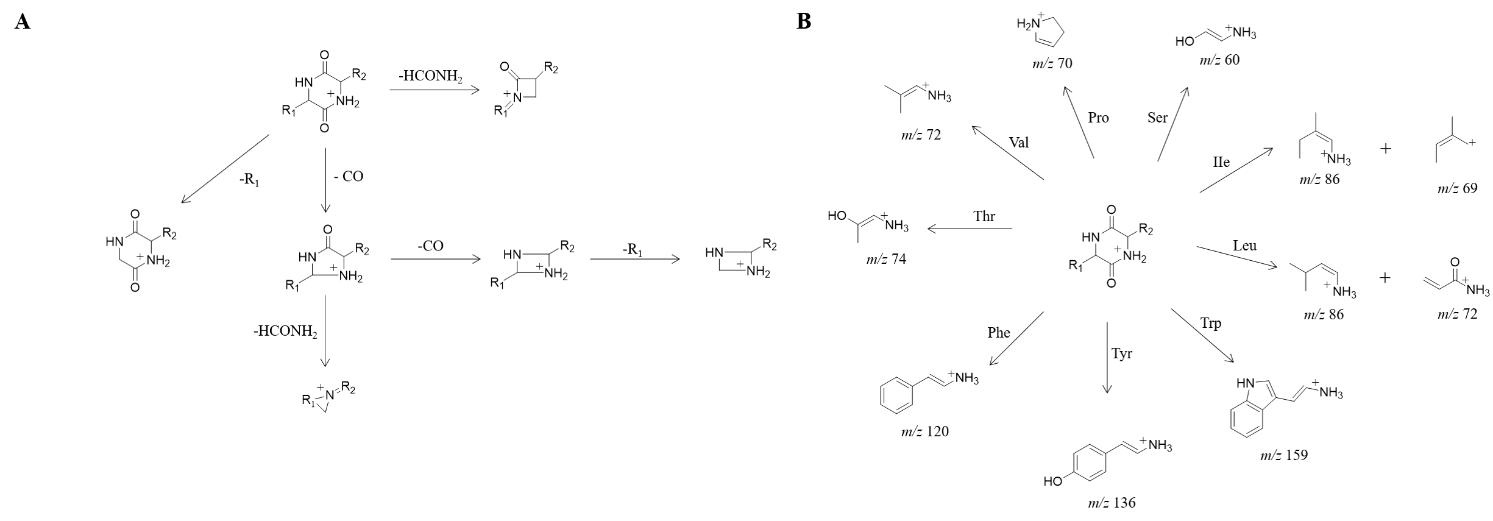


**Supplementary Figure 2**. The proposed fragmentation of skeleton and amino acid residues in cyclic dipeptide. (A) The proposed fragmentation of cyclic dipeptide skeleton. (B) The proposed fragmentation of amino acid residues in cyclic dipeptide.

# Supplementary Table

## Molecular docking

The key docking parameters of the receptor protein are as follows.

| Target | PDB ID | Radius | Coordinates |
| --- | --- | --- | --- |
| SRC | 2BDJ | 10.209 | 17.762052 5.447085 24.835189 |
| EGFR | 5UG9 | 9.375 | -13.812945 15.013361 -26.627131 |
| ESR1 | 5ACC | 10.514 | 14.163503 22.231070 65.014158 |
| PTGS2 | 5KIR | 6.524129 | 23.307477 1.314592 34.491219 |
